# Antioxidant Drug Therapy Approaches for Neuroprotection in Chronic Diseases of the Retina

**DOI:** 10.3390/ijms15021865

**Published:** 2014-01-27

**Authors:** Andrew J. Payne, Simon Kaja, Yuliya Naumchuk, Nancy Kunjukunju, Peter Koulen

**Affiliations:** 1 Vision Research Center, Department of Ophthalmology, School of Medicine, University of Missouri—Kansas City, Kansas City, MO 64108, USA; E-Mails: paynea@umkc.edu (A.J.P.); kajas@umkc.edu (S.K.); yn8r4@umkc.edu (Y.N.); kunjukunjun@umkc.edu (N.K.); 2 Department of Basic Medical Science, School of Medicine, University of Missouri—Kansas City, Kansas City, MO 64108, USA

**Keywords:** reactive oxygen species, oxidative stress, neuroprotection, age-related macular degeneration, diabetic retinopathy, glaucoma

## Abstract

The molecular pathways contributing to visual signal transduction in the retina generate a high energy demand that has functional and structural consequences such as vascularization and high metabolic rates contributing to oxidative stress. Multiple signaling cascades are involved to actively regulate the redox state of the retina. Age-related processes increase the oxidative load, resulting in chronically elevated levels of oxidative stress and reactive oxygen species, which in the retina ultimately result in pathologies such as glaucoma or age-related macular degeneration, as well as the neuropathic complications of diabetes in the eye. Specifically, oxidative stress results in deleterious changes to the retina through dysregulation of its intracellular physiology, ultimately leading to neurodegenerative and potentially also vascular dysfunction. Herein we will review the evidence for oxidative stress-induced contributions to each of the three major ocular pathologies, glaucoma, age-related macular degeneration, and diabetic retinopathy. The premise for neuroprotective strategies for these ocular disorders will be discussed in the context of recent clinical and preclinical research pursuing novel therapy development approaches.

## 1. Introduction

The largest risk factor for ocular and retinal disease and loss of vision remains age. Current theories of how aging affects cellular viability assume that oxidative damage accumulates with time and triggers downstream pathologies commonly associated with aging [1,2].

The visual signal transduction cycle is a high energy demanding function that results in a high metabolic rate of neurons and other cell types of the retina. The redox state of the retina is actively regulated by multiple signaling cascades that are optimized to function in concert with the oxidative load required to drive the visual cycle [3]. With aging, the oxidative load of ocular tissues increases and the antioxidant systems degrade to the point that retinal pathologies such as glaucoma or age-related macular degeneration (AMD) develop [3]. Endogenous oxidative stress associated with aging is weighed against the cell’s ability to mitigate the production of reactive oxygen species (ROS), such as free radicals or hydroxyl radicals, as part of normal respiratory function [3]. The main retinal source of ROS are mitochondria as part of the production of ATP by the classical glucose pathway, but mitochondria also regulate the intracellular pH, calcium concentration, and contribute to apoptotic signaling pathways [3]. A non-mitochondrial source of ROS has been discovered in photoreceptors, where components of the electron transport chain were identified in the outer segment disks of rod photoreceptors [4,5]. However, the contribution of such mechanism to energy metabolism and ROS generation in ocular pathologies remains to be elucidated further.

Electron leakage from complexes I and III in the glucose pathway result in the intracellular production of superoxide (O_2_·−) [6], which is then converted to hydrogen peroxide (H_2_O_2_) by the scavenger enzyme superoxide dismutase (SOD), specifically its two isoforms, Cu/Zn SOD (SOD1) in the cytosol, and Mn SOD (SOD2) in the mitochondrial matrix [7]. Hydrogen peroxide is unstable, but its reaction with reduced iron (Fe^2+^) yields the hydroxyl radical (OH·), a particularly potent ROS that is known to modify proteins, catalyze the formation of membranous lipid peroxyl radicals, and mutate DNA bases with a cyclization reaction [7].

Neuroprotective approaches to treating retinal pathologies must counter the toxic chemistry resulting from the generation of ROS, but should also have the ability to restore normal cell function. For example, *N*-acylethanolamines (NAEs) have been tested both *in vitro* and *in vivo* for efficacy as a neuroprotectant. NAEs are endogenously generated as part of lipid signaling pathways in the central nervous system (CNS) [8] including the retina [9]. Protective NAEs have been found to increase during periods of injury or oxidative stress, a cellular self-defense property that decreases with age [8,10]. While NAEs bind cannabinoid receptors (CB1 and CB2) [8], hence the name “endocannabinoids”, the neuroprotective effects are not cannabinoid mediated [10,11]. Different NAE species have been tested in the middle cerebral artery occlusion (MCAO) rat stroke model and have been found to reduce stroke volume and improve behavioral outcomes [10,12]. Neuroprotective effects of NAEs are not affected by blockade of CB1 or transient receptor potential vanilloid 1 (TRPV1) receptors but the cannabinoid uptake inhibitor AM404 blocked the neuroprotective effects of NAEs [10]. Further *in vitro* studies indicated that the NAE neuroprotective effect was mediated by an intracellular mechanism due to the reduction of oxidative damage and glutamate excitotoxicity through control of intracellular calcium concentrations [11,13]. NAE 18:2 was also found to block glutamate excitotoxicity in *ex vivo* retinal explants [14]. NAEs acting on non-cannabinoid targets lack the side effects often associated with cannabinoid related compounds [9]. This makes NAEs safe candidates for further study in the neuroprotection of ophthalmic disorders and provides an example for the need to comprehensively assess signaling pathways affected by both pharmacology and involvement in disease physiology of potential drug candidates.

Oxidative stress causes profound damage to the retina through dysregulation of intracellular physiology leading to neurodegenerative disorders. Below we will discuss the involvement of oxidative stress in each of the three major ocular pathologies characterized by chronic neurodegeneration, glaucoma, age-related macular degeneration (AMD), and diabetic retinopathy (DR). Recent clinical and preclinical research to discover novel therapies for these blinding diseases will also be discussed.

## 2. Age-Related Macular Degeneration (AMD)

During the normal function of the visual cycle, parts of photoreceptor outer segments (POSs) are shed towards the retinal pigment epithelia (RPE) layer for degradation [15]. This restorative function of the RPE is a process with a high energy demand intrinsic to the function of the retina, requiring extensive metabolic support through Bruch’s membrane (BrM) lining the RPE. BrM is a dense extracellular matrix that serves as a filter for the choriocapillaris (CC) and contributes to the immune privilege that separates the retina from the blood supply [15]. The filtering ability of BrM is directly proportional to the capacity of the RPE layer to process POSs since it mediates the exchange of nutrients from the blood for waste products from the RPE. An interruption in this physiological process contributes to AMD. The initial dysregulatory event of the POS/RPE/BrM/CC interaction is no small area of debate for obvious reasons [15].

Multiple sources of oxidative stress, aging, and genetic factors potentially contribute to AMD as a disease state arising from these multiple sources of oxidative stress associated with aging. AMD has no conclusive biomarker but it is easily diagnosed structurally by fundoscopic determination of large drusen (>125 microns), drusen, autofluorescent lipofuscin granules, and loss of RPE [16]. Distinct and circumscribed areas of RPE loss is an end stage marker for dry AMD known as geographic atrophy (GA). Whether RPE loss presages or results from the deterioration of the underlying choroid capillary bed is debatable [15], but the patchy loss of RPE is a clinically diagnosable factor. Fifteen percent of AMD cases will progress to the ‘wet’ variety where newly synthesized developing blood vessels invade the retina, known as choroidal neovascularization (CNV), to supply a nutrient deficit to the RPE [17]. CNV capillaries typically cause bleeding under the layers of the RPE, which then progresses to bleeding in the subretinal space. Occasionally, bleeding into the vitreous can occur before the vessel epithelia die, leaving disciform scars on the surface of the retina. Interestingly in the case of CNV type 1, the RPE layer is maintained by *de novo* generation of capillaries, while CNV type 2 destroys the RPE and invades into the subretinal space [15].

Several markers of cellular oxidative stress fundamentally characterize AMD. Oxidative modification of DNA has been observed in both genomic and mitochondrial DNA [18–20]. Lipofuscin granules are intracellular photoreactive aggregates in the RPE composed of pigment remnants [21]. Blue light excitation of lipofuscin granules excites the bisretinoid A2E, a condensation molecule of two all-trans-retinaldehyde and one phosphatidylethanolamine [22], to generate intracellular cytotoxic ROS [23,24]. Lipofuscin granules have been shown to produce singlet oxygen, superoxide, hydrogen peroxide, and increased lipid peroxidation [25]. It should be noted that the total ROS produced by the photo-excitation of lipofuscin granules is at least an order of magnitude greater than the ROS attributable to A2E [26], meaning the less well characterized constituents of lipofuscin are active photo-oxidizers [22]. Drusen are accumulations of lipid enriched material that are one of the categorical hallmarks of AMD [27]. Donor eyes positive for both drusen and basal linear deposits were found to be 24 times more likely to be positive for AMD [28]. Accumulations of drusen are thought to be a result or potentially also causative of decreased passive diffusion of RPE material through BrM. Proteomics studies of drusen have found them to be enriched with POS proteins and POS degradation products [29]. Proteins from drusen were also rich in posttranslational oxidative modifications such as carboxyethylpyrrole (CEP) protein adducts and advanced glycosylation end products (AGEs) [29]. CEP protein adducts are generated from the oxidation of docosahexaenoic acid (DHA), a polyunsaturated fatty acid (PUFA) found principally in the photoreceptors [15]. In a recent study, CEP labeled serum albumin was injected into mice and was found to recapitulate a dry AMD phenotype [30]. AGEs are carbohydrate posttranslational protein modifications that are accumulated over a lifetime and activate the aptly named receptor of AGE (RAGE) to initiate an inflammatory response [31].

### Pharmacologic Treatments for AMD

Previous to the new millennium, the most common treatment modality for wet AMD was the use of a laser to cauterize emergent blood vessels [16]. While laser treatment is efficacious in preventing further vision loss due to the particular cauterized capillary, the CNV process proceeds apace. New treatments for wet AMD reached the clinic in the mid-2000s and now wet AMD is fairly well controlled by treatment with anti-vascular endothelial growth factor (VEGF) antibodies ranibizumab or bevacizumab, the anti-VEGF agent aflibercept that contains portions of the VEGF-binding domains of the human VEGF receptors 1 and 2 or the polyethylene glycol modified RNA aptamer pegaptanib [16]. Anti-angiogenic therapies block the chemoattractant and blood vessel growth stimulating effects of VEGF to prevent vision loss associated with CNV scaring on the retina. This treatment modality however only prevents further vision loss but does not address the underlying pathology that leads to the stimulation of CNV [32]. A vaccine composed of a peptide segment of the VEGF receptor 2 (VEGFR2_400–408_) has been demonstrated to reduce CNV related lesion size in mice [33,34]. A Phase I clinical study of the VEGFR2_400–408_ vaccine was recently completed (Anti-VEGFR Vaccine Therapy in Treating Patients With Neovascular Maculopathy; NCT00791570) but results have not yet been reported.

Treatment of dry AMD has proven more difficult. The Age-related Eye Disease Study (AREDS) was a multicenter examination of antioxidant vitamin supplementation in the pathogenesis and advancement of AMD. Almost 5000 AMD patients were enrolled into one of four categories of increasing AMD severity and treated with an oral vitamin supplement combination of vitamins C and E, β-carotene, zinc oxide, and cupric oxide [17]. The AREDS formulation was only beneficial to the most advanced AMD cases, those patients in categories 3 and 4 where the AREDS formulation decreased the probability of visual acuity decrease by 19% [16,17]. The AREDS formulation seems to have also slowed progression to more advanced AMD by 25% for those that presented with intermediate or advanced AMD [16,17]. There was no measurable effect on groups 1 or 2, by far the largest group in the public, to the more advanced stages of AMD (categories 3 and 4) [17,35,36]. Due to the disappointing results of the AREDS, AREDS2 was initiated in 2006. AREDS2 modified the original AREDS formulation by replacement of β-carotene with lutein and zeaxanthin, decreased zinc, and added the ω-3 long chain PUFAs DHA and eicosapentaenoic acid (EPA) [37,38]. The initial AREDS2 report indicated no improvement to any visual parameter measured when compared to the standard AREDS formulation, although there was a decrease of an already insignificant coincidence of lung cancer [38]. It is not clear if this risk of lung cancer is not due to a confounding factor like former smoking habits of the patients. Smoking is the second most common cause of AMD, the most common cause is aging, [16,39] and 91% of the 31 lung cancers that developed during the AREDS2 study were former smokers [38]. Alternatively, the β-carotene in the AREDS formulation could also contribute to the lung cancer but that hypothesis requires further evaluation [38].

Small molecule antioxidant treatments for dry AMD have been proven effective preclinically *in vitro* but have yet to be tested in clinical trials. Agonists of the 5HT-1 serotonin receptor AL-8309 and 8-OHDPAT were shown to be neuroprotective in rodents. AL-8309 and 8-OHDPAT countered a phototoxic insult in rats, the protective effect was abolished by pretreatment with the 5HT-1A antagonist WAY-100635 [40]. AL-8309 was also demonstrated to block the activation of microglia following phototoxic insult and inhibit the deposition of complement factor proteins at the lesion site [41]. 8-OHDPAT was also found to decrease lipofuscin aggregation, counter hydrogen peroxide insult, and increase expression levels of glutathione and MnSOD2 [42]. These compounds are potential treatments for the chronic condition of dry AMD, but have yet to enter clinical trials. Recently, phase I clinical trial results for emixustat hydrochloride (ACU-4429; Acucela Inc., Seattle, WA, USA), an inhibitor of the RPE-specific 65 kDa protein isomerase (RPE65) with *in vitro* efficacy at reducing A2E accumulation, were reported [43,44]. Daily treatment was tolerated with 67% of patients reporting adverse effects, most commonly chromatopsia that resolved upon the cessation of treatment [43,44]. Future trials will determine the potential therapeutic benefit of emixustat for prolonging or maintaining visual function in AMD.

Two derivatives of TEMPOL, a superoxide dismutase mimetic, are potential neuroprotective antioxidants. OT-647 reduced the oxidative burden produced by lipofuscin excited at 430 nm in cultured ARPE-19 cells [45]. OT-647 had greater efficacy when compared to Trolox or alpha-tocopherol (vitamin E) [45]. OT-551 is a compound similar to OT-647 with improved efficacy at prevention of phototoxic damage and inhibition of PUFA oxidation products in rats [46,47]. A small Phase II clinical trial of OT-551 in 10 patients with geographic atrophy (GA) arrested decreases in visual acuity when compared to the untreated control eye but did not alter the area of GA or total drusen area [48]. A static treatment to prevent AMD progression may be an acceptable endpoint, OT-551 may have greater efficacy than the AREDS antioxidant formulation to halt the progression of AMD, but this compound needs to be clinically validated.

Rapamycin (Sirolimus) is a well-known immunosuppressant and anti-inflammatory that inhibits the mammalian target of rapamycin (mTOR) protein [49]. Sirolimus was found to decrease complement accumulation and CNV through a non-VEGF mechanism in a mouse model [50]. Sirolimus has been tested in a small Phase I/II trial as an adjuvant treatment with anti-VEGF therapy (as needed; bevacizumab or ranibizumab at the treating physician’s discretion) in cases of CNV [51]. The study only enrolled 13 patients but demonstrated that co-treatment with an anti-angiogenic and an immunosuppressant decreased the required number of anti-angiogenic injections by approximately 50% [51]. However, Sirolimus failed a Phase I/II clinical trial for GA [52]. Sirolimus was hypothesized to treat GA because of its anti-inflammatory properties, but GA was worsened in the study eye *vs.* the control eye [52]. Visual acuity decreased by 21 letters in the study eye *vs.* only 3 letters decreased in the control eye [52]. Rapamycin is a viable co-treatment to treat wet AMD but failed as a solo treatment for advanced dry AMD.

Effective pharmacologic treatments for dry AMD are still lacking in the clinic. The AREDS antioxidant formulation has some efficacy in a subset of cases with a low frequency of side effects. Novel targets and a better mechanistic understanding of the disease are required for more efficacious treatment of dry AMD. The more aggressive and progressive form, wet AMD, has efficacious clinical treatments available in the form of anti-VEGF therapies attenuating neovascularization. While disease causing mechanisms are not eliminated, damage-inducing neovascularization is inhibited with continuous proper treatment.

AMD has many obvious markers of oxidative damage as part of its pathology hallmarks. As such, preventive antioxidant therapy has the potential to halt or slow the progression of disease with similar efficacy as other antioxidants are able to arrest other aspects of cellular aging. Future treatments may include direct antioxidants, but the most promising current treatments are those that both prevent cell death and restore homeostatic function. An efficacious neuroprotective treatment will likely consist of a drug with specific targets that directly affect critical clinical outcome measures.

## 3. Diabetic Retinopathy

Diabetic retinopathy (DR) is a common consequence of poorly controlled diabetes. Diabetes is associated with a 90% risk of DR onset within 25 years of diagnosis [53]. The major driving force of both neuronal and vascular damage in DR is systemic hyperglycemia resulting in pericyte cell death, thickening of the capillary basement membrane, and accumulation of advanced glycation end-products (AGEs) [53]. Hyperglycemia affects several major systems that ultimately culminate in the generation of toxic levels of oxidative stress including accumulation of AGEs and activation of the poly(ADP-ribose) polymerase (PARP), protein kinase C (PKC), hexosamine, and polyol pathways [54].

Oxidative stress has both direct and indirect effects on retinal neuronal degeneration. Reactive oxygen species (ROS) are major contributors to oxidative stress, which are responsible for the initiation of apoptosis and inflammatory pathways, eventually leading to angiogenesis and the destruction of neuronal tissue [53,54]. In turn, neuronal degeneration promotes even more apoptosis and inflammation in the retina [53,54]. It is these mechanisms that create a positive feedback loop responsible for the progression of DR as a complication of diabetes [53,54]. Oxidative stress also disturbs intracellular calcium homeostasis [54–57] and causes damage by neuronal hyperexcitability [56], resulting in further neuronal sensitization to the deleterious effects of hyperglycemia and weakening of cellular self-defense mechanisms against endogenous and exogenous insult.

One major pathway by which hyperglycemia contributes to neurodegeneration is through the generation of AGEs, which are produced from glycating dicarbonyl compounds. Chronic hyperglycemia creates a favorable environment for non-enzymatic condensation reactions between reduced glucose and amine residues of proteins, nucleic acids, and lipids resulting in an irreversibly cross-linked, complex of compounds collectively termed AGEs (for review, see [54,58,59]). Elevated levels of AGEs have been found in retinal blood vessels, serum, and vitreous of diabetic patients and contribute to DR pathophysiology mainly by disturbing microvascular homeostasis through binding of AGEs to the protein, receptor of AGE (RAGE). AGEs accumulate in pericytes, causing damage to endothelial cells and contributing to blood–retinal barrier dysfunction [54,59,60]. AGEs also cause endothelial cells to express different adhesion and chemoattraction factors through intracellular ROS generation [54]. Furthermore, AGEs activate nuclear factor-κB (NFκB) and nicotinamide adenine dinucleotide phosphate (NADPH) oxidase, which in turn increases ROS production and apoptosis of retinal neurons [54,61]. Therefore, targeting and disrupting the AGE-RAGE interaction represents a potential therapeutic intervention strategy, which could eliminate many of the secondary pathways affecting cellular viability and resulting from increased AGEs levels, as shown in preclinical models [54].

Another molecule that contributes significantly to both neuronal and vascular degeneration is poly(ADP-ribose) polymerase (PARP), which becomes activated in the retina of diabetic animals [54]. Excessive amounts of PARP will bind NAD^+^ and ultimately result in decreased glycolysis and eventually cell death. Secondly, PARP inhibits glyceraldehyde-3-phosphate dehydrogenase (GAPDH), further increasing ROS and reactive nitrogen species (RNS) production, DNA strand breaks, and endothelial and neuronal dysfunction in DR [54]. Administration of PARP inhibitors in preclinical animal models resulted in decreased oxidative damage to the retina and reduced glial activation, resulting in reduced severity and progression of DR [54,62,63].

One major pathological hallmark of diabetes is diminished levels of neurotrophic factors [64,65]. Loss of trophic support compromises neuronal regeneration potentially resulting in an increased susceptibility to damage and irreversible pathological neurodegeneration. Several neurotrophic factors have been implicated in the pathophysiology of DR, including insulin, pigment epithelium-derived factor (PEDF), nerve growth factor (NGF), and brain-derived neurotrophic factor (BDNF) [54]. For example, BDNF, a growth factor responsible for neuronal growth and survival, is decreased in patients with type 2 diabetes mellitus (T2DM), indicative of insulin sensitivity [64,65]. Further study of neurotrophic factors and their role in aging and disease pathologies has the potential to ultimately reveal mechanisms that may be significant and vital for preventing the degenerative changes caused by DR and other neurodegenerative diseases. In addition, clinical data show that neurotrophic factors could potentially serve as biomarkers for DR, but current evidence for their therapeutic potential is limited [54].

### 3.1. Oxidative Stress in DR

Strong evidence accumulated over the past 25 years of research indicates correlations between elevated levels of oxidative stress and physiological and molecular mechanisms of oxidative stress in the DR patient [66]. From this, it has been established that oxidative stress is critical in the pathophysiology of DR [66]. These findings include evidence of mitochondrial DNA (mtDNA) damage, serum and plasma markers for oxidative stress, as well as changes in antioxidant enzymes, and the presence of elevated ROS in both neuronal and vascular tissues [66].

Oxidative stress has been shown to contribute to mtDNA damage by causing increased formation of 8-hydroxy-2′-deoxyguanosine (8-OHdG), resulting in increased mtDNA deletions [67]. This early study reported an increase of delta mtDNA4977 deletions and 8-OHdG modifications in muscle mtDNA of diabetic patients [67]. Increased mtDNA damage was positively correlated with the severity of DR, an indication of the importance of oxidative stress in the progression of DR [67]. *In vitro* studies have further shown that initial mtDNA damage leads to further ROS production, thereby initiating a vicious cycle that is responsible for the progression of DR pathology [68].

In addition, there is evidence of increased levels of oxidative stress in the blood of DR patients. In 2000, a clinical study compared the serum levels of superoxide dismutase (SOD) and glutathione (GSH) in patients with type 1 diabetes mellitus (T1DM) and type 2 (T2DM) with those of healthy subjects. Decreased levels of SOD and GSH in both T1DM and T2DM patients provided strong evidence that oxidative stress potentially contributes to the pathogenesis of diabetes mellitus [69]. A correlation between SOD levels and disease progression has also been detected in diabetic patients [70]. Furthermore, T2DM patients with DR had elevated serum levels of a malondialdehyde (MDA)-like metabolites when compared to T2DM patients without angiopathic complications, thus demonstrating a significant correlation between serum lipid peroxidation and duration of the disease [69]. Another clinical trial not only confirmed the correlation between lipid peroxidation and disease duration but also stated that the very same correlation exists between lipid peroxidation and disease severity [70]. As a marker for lipid peroxidation, the MDA metabolite could potentially serve as a marker for the DR severity [70]. Most recently, serum MDA levels were found to be significantly higher in proliferative DR (PDR) *vs.* non-proliferative DR (NPDR) patients [71].

When comparing patients with PDR and NPDR there was no significant difference detected in the serum levels GSH, nitric oxide (NO), and copper in these two groups [72]. In contrast, the advanced oxidation protein product (AOPP) concentration was found to be much higher in patients with PDR when compared to healthy subjects [72]. These findings corroborate the hypothesis that hyperglycemia causes accelerated non-enzymatic glycosylation and oxidative stress [72]. Intensive research efforts have since utilized serum AOPP levels in an attempt to gain more knowledge on the connection between oxidative stress and DR pathogenesis. A 2008 study showed that AOPP, protein carbonyl, and 8-OHdG levels were significantly elevated in diabetic patients with DR when compared to diabetic patients without retinopathy [73] although these results were not confirmed by another study [74], a discrepancy that is likely due to the still incomplete diagnostic assessment of early-stage DR. While AOPP levels are correlated with lipid peroxidation, there is some discrepancy to what extent AOPP levels are predictive of DR. These new findings confirmed that severe lipid peroxidation, protein oxidation, and oxidative DNA damage result from diabetes, and that the severity of diabetes directly correlates with disease progression and microvascular complications [73]. It is, therefore, safe to assume that oxidative stress is a significant risk factor for the development of DR and that the levels and types of serum ROS and oxidative stress byproducts can potentially become helpful markers in predicting the severity of retinopathy [73] and provide potential targets for drug development.

Additional potential serum markers have recently been tested in clinical studies for DR. Serum levels of high-density lipoprotein (HDL)-associated paraoxonase (PON1) were significantly decreased in DR patients in comparison to the control group [75]. Similarly, ferric-reducing ability of serum (FRAS) levels in DR patients were not only significantly decreased in comparison to serum levels in the control group, but were also significantly lower than in diabetic patients without retinopathy [75]. These findings strengthen the notion of oxidative stress as the critical factor underlying the pathophysiology of DR. Interestingly, the ratio of PON1:C-reactive protein (CRP) in DR patients, diabetic patients without DR and healthy control subjects was highly correlated with the clinical diagnosis and disease severity, making the PON1:CRP ratio a potential prognostic marker for the progression of DR [23].

### 3.2. Approaches for Neuroprotection in DR

Neuronal changes in DR precede vascular changes, indicating that the initial retinal dysfunction is caused by early neurodegenerative rather than vascular events [54,76,77]. The pathogenesis is initiated by hyperglycemia that disrupts retinal glutamate homeostasis and results in excitotoxicity [77–79]. Pathologically elevated extracellular glutamate chronically activates NMDA receptors causing an influx of extracellular calcium, ultimately resulting in intracellular free radical formation and apoptosis [80]. Other known physiological processes that contribute to retinal cell death are glial cell activation and altered function of neurotrophic factors, exacerbating the neuronal damage in the retina [81,82]. The fact that neurodegeneration precedes vascular damage as an initial event in DR provides both rationale and premise for neuroprotection studies in DR.

While the initial biochemical changes are clinically silent in the beginning of DR disease development, damage at the cellular level is irreversible and difficult to treat [76,77,82]. Once DR progresses to the point when clinical symptoms become apparent the damage is typically extensive and irreversible [76,77,82]. Treatment options are limited to surgical intervention in form of laser coagulation therapy targeting vascular damage and a few pharmacological supplements. Currently, maintenance of good glycemic control still remains the only available strategy in preventing DR and delaying DR progression [83].

The more we learn about the pathophysiological mechanisms of DR, the more evident it becomes that multiple cellular signaling pathways, reactions, and molecules involved in the pathogenesis of DR are targets that need to be evaluated for their potential therapeutic benefit, either as single targets or for the development of combination therapies. The ability to monitor neuronal changes in diabetic patients could allow the earlier detection of developing DR and provide an opportunity for earlier treatment regimens that open wider therapeutic opportunities and potentially better outcomes. Biomarkers for disease progression, therefore, are needed to detect the clinically silent period preceding the occurrence of symptoms and for monitoring the underlying condition.

Despite good evidence for early onset of neurodegeneration in DR prior to vascular damage there have been only a few clinical trials focusing on neuroprotection and neurodegeneration in DR recently. A clinical trial that evaluated the effect of antioxidant therapy on DR patients after a 5-year follow-up period was published in 2011 [84]. The study found that antioxidant therapy was not effective at improving best corrected visual acuity, however, antioxidant supplementation showed a statistically significant retardation of progression as well as maintenance of antioxidant plasma levels [84]. (−)-Epigallocatechin gallate from green tea has been demonstrated as having neuroprotective properties in the retina [85], although only in moderate doses [86]. In 2012, the results of two large trials for fenofibrate, a fibric acid derivative that activates peroxisome proliferator-activated receptor alpha, were published: the FIELD (Fenofibrate Intervention and Event Lowering in Diabetes) and ACCORD (Action to Control Cardiovascular Risk in Diabetes)-Eye studies [87]. In both studies fenofibrate was effective at reducing the progression of neurodegeneration, with patients with preexisting DR benefitting more from the drug treatment [87]. Furthermore, fenofibrate reduced the need for a first laser treatment by 31% and resulted in an overall reduction of DR by 5% over the five years in the FIELD study and by 3.7% over the four years in the ACCORD-Eye study [87]. While the mechanism of action of fenofibrate in DR has not yet been elucidated, it appears that both lipid and non-lipid pathways are involved in the reduction of apoptosis, oxidative stress, and inflammation [87]. Overall, both studies concluded that fenofibrate could be a useful, neuroprotective treatment in early diabetic neuropathy [87].

Altogether, there is evidence that antioxidant and neuroprotection strategies are able to benefit patients with DR. Given the potential of antioxidant and neuroprotectant supplementation to slow progression of DR, new clinical trials should include combination therapies.

## 4. Glaucoma

Glaucoma is a group of multifactorial ocular disorders characterized by atrophy of the optic nerve and degeneration of retinal ganglion cells (RGCs), ultimately leading to partial or complete vision loss [88]. At present, glaucoma is the world’s second leading cause of blindness and the leading cause of irreversible vision loss [89,90]. Existing pharmacotherapies targeting ocular hypertension are largely unsatisfactory and can only slow disease progression [91]. It should be noted that elevated intraocular pressure (IOP), potentially originating from reduced outflow via the trabecular meshwork and/or the uveoscleral pathway, is the most important biomarker for glaucoma. Controlling ocular hypertension is currently the only approach for pharmaceutical intervention, however, the hypothesis that elevated IOP is the determining factor for glaucoma has been almost universally rejected [92], as patients with normal IOP can show damage typical for glaucoma and as individuals with elevated IOP may display no evidence of optic neuropathy [93].

Multiple mechanisms are involved in the pathology of glaucoma that ultimately culminate in RGC death and the subsequent loss of vision. These mechanisms include chronic or intermittent ischemia, excitotoxicity, loss of trophic factor supply and synaptic dysfunction [88,89,91]. Intriguingly, many of these disease pathways are a direct consequence of, or will ultimately result in, elevated levels of oxidative stress within the tissues affected by early stage glaucoma. For instance, ischemia leads to an unbalanced metabolic demand of cells which results in the generation of free radicals and ROS [94]. Excitotoxicity can reduce the activity of the cystine/glutamate antiporter resulting in the depletion of intracellular glutathione and subsequent cell death by oxytosis, a programmed cell death pathway induced by oxidative stress [95,96]. Similarly, mitochondrial dysfunction as a result of synaptic aging and the subsequent loss of trophic factor supply due to diminished axonal transport results in elevated levels of oxidative stress, reduced antioxidant enzyme levels, and oxidative damage of mtDNA.

Clinical evidence for oxidative stress in glaucomatous tissue generates the premise and is associated with challenges for the development of neuroprotection strategies to treat glaucoma.

### 4.1. Evidence for Oxidative Stress in Glaucoma

Accumulating evidence points to a causal role of elevated levels of oxidative stress inherent to glaucomatous neuropathies. A number of clinical studies have shown increased oxidative stress, oxidative DNA damage, and mitochondrial dysfunction in tissues affected by glaucomatous neuropathies.

Some of the earliest evidence of oxidative stress was derived from the detection of endothelial leukocyte adhesion molecule-1 (ELAM-1) in trabecular meshwork (TM) cells in human glaucomatous eyes [97]. ELAM-1 is a diagnostic marker for atherosclerotic plaques in the vasculature and represents a protective cellular response against oxidative stress.

This initial data led to the investigation of oxidative DNA damage in human TM. The development of a biochemical method to analyze 8-OH-dG, as correlate for mtDNA damage in human TM biopsy tissue, enables the identification of glutathione *S*-transferase isoenzymes’ (GSTM1 and GSTT1) activity in human TM [98]. The levels of 8-OH-dG were significantly increased in glaucoma patients and oxidative DNA damage was highly correlated with intraocular pressure (IOP) and visual field defects [98]. Furthermore the GSTM1-null genotype was associated with higher 8-OH-dG levels and statistically significantly more common in patients with primary open-angle glaucoma (POAG) when compared to control subjects [98]. In a separate study, statistically significant correlations between oxidative damage in human TM and visual field damage and IOP were reported [99]. More recently, the increased incidence of the GSTM1-null allele in subjects with POAG was confirmed by another group, however, no statistical significance could be ascertained, likely due to the small sample size [100].

The total reactive antioxidant potential (TRAP) was significantly reduced in the aqueous humor of POAG patients, while glutathione generation was increased threefold [101], suggesting that oxidative stress resulting from mitochondrial dysfunction leads to an increase in antioxidant enzymes and contributes to an overall reduction in TRAP [101].

Nitrotyrosine immunostaining of post-mortem biopsies of the pre-laminar optic nerve head, revealed peroxynitrite-mediated injury of endothelial and smooth muscles cells, as well as of astrocytes [102]. Immunoreactivity for nitrotyrosine correlated positively with disease progression and was significantly higher in glaucomatous tissue when compared with tissue from normal control eyes [102]. Similar evidence for increased levels of oxidative stress was identified in human glaucomatous lamina cribrosa cells based on quantification of ROS production using the thiobarbituric acid reactive substances (TBARS) assay [103]. Lamina cribrosa cells from glaucomatous eyes exhibited mitochondrial dysfunction and impaired calcium extrusion compared to lamina cribrosa cells from healthy donors [103].

More recently, several studies have focused on biomarker identification and analysis in glaucoma. Serum from patients with glaucoma and healthy control subjects was analyzed in a multicenter case control study for a number of oxidation degradation products, antioxidants, vitamin E, and antioxidant enzymes [104]. While all potential biomarkers were statistically significantly different between glaucoma patients and healthy subjects, the authors noted large variability for all parameters. However, vitamin E was identified as the most consistent biomarker, which was increased in the glaucoma patient group and correlated significantly with clinical hallmarks of disease progression, including IOP and visual acuity [104]. This study highlights the difficulty of identifying specific blood biomarkers for glaucoma and the need for novel disease-specific biomarker panels. However, blood analysis has added to the substantial body of evidence for oxidative stress in glaucoma. A recent study investigated TRAP and superoxide dismutase (SOD) as indicators of antioxidant status, the total oxidant status and several indicators of oxidative stress (NO, protein carbonyl and MDA) in serum of glaucoma patients [105]. Of particular interest, strong evidence of decreased antioxidant defense and increased oxidative stress levels were found in both POAG and pseudoexfoliative glaucoma [105], indicating potentially the same role for oxidative stress in different etiologies of the disease. Furthermore, oxidative stress has been identified in serum of patients with primary angle-closure glaucoma [106].

In summary, there is a large body of clinical data that supports a role of oxidative stress and mitochondrial dysfunction in the etiology of glaucoma pathologies.

### 4.2. Neuroprotection as a Therapy Approach for Glaucoma

Neuroprotective strategies for glaucoma are designed to either slow the progression of glaucomatous neuropathy by either delaying or preventing RGC loss. Given the involvement of oxidative stress in the pathophysiology of glaucoma, neuroprotectants must be tested for their ability to either increase cellular resistance to the deleterious effects of oxidative stress or to target oxidative stress itself by using drug candidates with antioxidant effects.

There are only a few preclinical studies investigating the beneficial effects of antioxidants in glaucoma, likely due to the lack of clinically relevant animal models for glaucoma that accurately recapitulate the etiology of human disease development and the great difficulties associated with long-term longitudinal studies. In addition, drug delivery to the neural retina as protection from or attenuation of pathological changes occurring during glaucoma still represents a major problem for glaucoma therapy development, similar to the CNS in general where difficulties with drug delivery result in major obstacles for the development of effective neuroprotective therapies.

Recently, the antioxidant α-lipoic acid was shown to protect RGCs, improve retrograde axonal transport in the optic nerve and increase antioxidant gene and protein expression using the DBA/2J mouse model [107]. Unfortunately, however, there are no functional data available with respect to visual performance, which can be measured in mice and is affected in the DBA/2J glaucoma model, as shown previously [108]. However, a recently published study using a rat model of glaucoma showed that 17β-estradiol containing eye drops provide effective neuroprotection and prevented a decline in visual acuity even in the presence of continually elevated IOP [109]. Estrogens have been shown to effectively protect the retina in preclinical animal models [110] and can act as direct free radical scavenging phenolic antioxidants [111].

One clinical Phase 3 study (“Impact of Oral Versatile Antioxidants on Glaucoma Progression”, NCT01544192) testing three antioxidants (α-tocopherol, Gingko biloba and an antioxidant formula) was completed last year, but results have not yet been reported.

Another drug development focus has been on neuroprotective agents. However, despite the success of neuroprotective strategies in preclinical models, essentially all of the more than 100 clinical neuroprotection trials for glaucoma have failed in Phase 2 or Phase 3 clinical trials due to failure of meeting the efficacy or patient safety standards.

The largest neuroprotection trial for glaucoma failed to show efficacy of memantine, an NMDA receptor antagonist. The details of the memantine trial by Allergan Inc. (Irvine, CA, USA; “Memantine in Patients With Chronic Glaucoma”, clinicaltrials.gov NCT00168350) have still not been reported, however, the drug failed to meet its primary endpoint and a second Phase 3 trial could not replicate the statistically significant effects of the drug on a secondary functional endpoint of an earlier trial.

Given the success of memantine as cognitive enhancer, which was FDA-approved in 2003 for cases of moderate to severe Alzheimer’s disease [112], and an extensive body of evidence showing successful neuroprotection in preclinical glaucoma studies [113–115], one has to ask the question why memantine failed in clinical trials. The greatest challenges faced when setting up clinical trials for neuroprotection are the choice of endpoint and the selection of trial subjects. While the success of most preclinical studies is determined by molecular or cellular outcome measures, human trial endpoints are critically dependent on functional improvement or an attenuation of the rate of decline of visual performance, both of which are long-term processes.

Therefore, novel strategies are needed that target multiple pathophysiological aspects that contribute to the disease phenotype, most importantly oxidative stress and excitotoxicity. This need is highlighted by the fact that memantine treatment can prevent neuronal necrosis induced by excitotoxicity in Alzheimer’s disease, but fails to reduce apoptosis from chronically elevated levels of oxidative stress [116]. A new clinical trial at the University Hospital of Angers (France) combining memantine with vitamin D (AD-IDEA trial; NCT01409694) is currently recruiting patients. The combination of the NMDA-receptor antagonist memantine with the neuroprotective, antioxidant and anti-inflammatory properties of vitamin D may indeed prevent neuronal loss in Alzheimer’s disease, but could also represent a promising strategy for glaucoma therapy [116].

Furthermore, novel compounds that are conjugates of memantine with antioxidants have shown promise *in vitro*. For example, α-lipoic acid covalently linked to memantine showed antioxidant activity *in vitro*, the ability to cross the blood brain barrier and to significantly inhibit β-amyloid(1–42) aggregation [117]. Given the beneficial effects of α-lipoic acid in the DBA/2J model for glaucoma [107] and the preclinical and clinical evidence and safety profile for memantine, this new compound has also promise as a strategy for glaucoma therapy. Similarly, medicinal chemistry has addressed such a combination therapy approach with novel compounds like bis(7)-tacrine (B7T), which consists of two tacrine moieties connected by a 7-carbon alkyl spacer group [118]. Tacrine is an inhibitor of acetylcholine esterase that has clinical utility in some cases of AD. However, B7T seems to generate neuroprotection as a NMDA receptor antagonist [119], blocking the excitotoxicity and downstream oxidative effects of influx of extracellular calcium [120]. RGC apoptosis after exposure to NMDA was decreased both in primary culture explants from neonatal rats and *in vivo* by co-application of B7T [121,122]. These preclinical studies of B7T indicate increased efficacy over memantine and decreased side effects when compared to delivery of tacrine alone, but this compound has yet to enter clinical trials.

In summary, there is strong preclinical evidence for the critical involvement of oxidative stress in the development of glaucoma, but there is also an urgent need for clinically relevant glaucoma models and animal studies testing the potentially beneficial effects of antioxidants on clinically relevant endpoints. However, even more important are new clinical trials with carefully chosen criteria for enrollment and well-defined endpoints that will test novel compounds and combination therapies of neuroprotectants with antioxidants in glaucoma.

## 5. Conclusions

Oxidative stress is the keystone in multiple lines of evidence converging on the origin and development of ocular disorders. The common source of the pathologies discussed above is oxidative stress, but it cannot be assumed that a single reductive or antioxidant treatment would be sufficient to reverse separate pathologies. Novel treatments must be devised with specific attention focused on the specific etiologies and pathophysiological mechanisms of each condition. Antioxidant treatments for glaucoma must address elevated IOP, RGC loss and optic nerve atrophy, while in AMD the deposition of lipofuscin or vascular pathologies must be corrected. Control of blood sugar is critical in diabetic patients to prevent DR as a complication and neuroprotective strategies must also be developed with potential vascular pathologies of DR in mind. The success of neuroprotective treatments of oxidative stress in ocular diseases affecting the retina depends largely on the careful execution of clinical trials, in particular with respect to the choice of endpoints, patient recruitment and enrollment and tightly controlled diagnostic criteria.
